# Meat and Fish as Sources of Extended-Spectrum β-Lactamase–Producing *Escherichia coli*, Cambodia

**DOI:** 10.3201/eid2501.180534

**Published:** 2019-01

**Authors:** Maya Nadimpalli, Yith Vuthy, Agathe de Lauzanne, Laetitia Fabre, Alexis Criscuolo, Malika Gouali, Bich-Tram Huynh, Thierry Naas, Thong Phe, Laurence Borand, Jan Jacobs, Alexandra Kerléguer, Patrice Piola, Didier Guillemot, Simon Le Hello, Elisabeth Delarocque-Astagneau, on behalf of the BIRDY study group

**Affiliations:** Institut Pasteur, Paris, France (M. Nadimpalli, L. Fabre, A. Criscuolo, B.-T. Huynh, D. Guillemot, S. Le Hello, E. Delarocque-Astagneau);; Institut National de la Santé et de la Recherche Médicale, Université de Versailles Saint-Quentin-en-Yvelines and Université Paris-Saclay, Paris (M. Nadimpalli, B.-T. Huynh, D. Guillemot, E. Delarocque-Astagneau);; Institut Pasteur du Cambodge, Phnom Penh, Cambodia (Y. Vuthy, A. de Lauzanne, M. Gouali, L. Borand, A. Kerléguer, P. Piola);; Assistance Publique/Hôpitaux de Paris, Bicêtre Hospital and Université Paris-Sud, Le Kremlin-Bicêtre, France (T. Naas);; Sihanouk Hospital Center of Hope, Phnom Penh (T. Phe);; Institute of Tropical Medicine, Antwerp, Belgium (J. Jacobs);; K.U. Leuven, Leuven, Belgium (J. Jacobs);; Assistance Publique/Hôpitaux de Paris, Raymond-Poincaré Hospital, Garches, France (D. Guillemot, E. Delarocque-Astagneau)

**Keywords:** ESBL, antibiotic resistance, food safety, lower- and middle-income countries, Southeast Asia, *Escherichia coli*, antimicrobial resistance, bacteria, extended-spectrum β-lactamases, Cambodia

## Abstract

We compared extended-spectrum β-lactamase–producing *Escherichia coli* isolates from meat and fish, gut-colonized women, and infected patients in Cambodia. Nearly half of isolates from women were phylogenetically related to food-origin isolates; a subset had identical multilocus sequence types, extended-spectrum β-lactamase types, and antimicrobial resistance patterns. Eating sun-dried poultry may be an exposure route.

In Europe, evidence for the spread of extended-spectrum β-lactamase (ESBL)–producing *Escherichia coli* from animals to humans via food is unclear (*1*). Few studies have been conducted in low- and middle-income countries, where colonization rates can exceed 60% (*2*). High ESBL colonization rates in low- and middle-income countries such as Cambodia are usually attributed to unrestricted consumer access to and hospital overuse of third-generation cephalosporins (*3*,*4*). However, antimicrobial drugs in classes critical for human health (e.g., β-lactams, macrolides, aminoglycosides, polymyxins) are increasingly being used in food animals (*5*). In Cambodia, weak public health protections and consumption of undercooked animal products could exacerbate the spread of ESBL-producing *E. coli* or ESBL genes from animals to humans.

We had 2 goals with this study. First, we assessed the prevalence of ESBL-producing or carbapenemase-producing *E. coli* from fish, pork, and chicken from markets in Phnom Penh, Cambodia. Second, we examined the contribution of food-origin isolates to locally disseminated ESBL-producing *E. coli* by comparing isolates from food with isolates from healthy, colonized persons and infected patients. 

## The Study

During September–November 2016, we purchased 60 fish, 60 pork, and 30 chicken samples from 150 vendors at 2 markets in Steung Meanchey district, Phnom Penh (Appendix Table 2) and tested them at the Institut Pasteur du Cambodge for third-generation cephalosporin- and carbapenem-resistant *E. coli* (Appendix sections 1.1–1.3). We detected ESBL-producing *E. coli* (all CTX-M-type) among 93 (62%) of 150 food samples, including 32 (53%) of 60 fish, 45 (75%) of 60 pork, and 16 (53%) of 30 chicken samples. We identified carbapenem-resistant *E. coli* (OXA-type) from 1 pork and 1 fish sample.

We also selected ESBL-producing *E. coli* from 88 recently pregnant healthy women living in Steung Meanchey and participating in the Bacterial Infections and antibiotic Resistant Diseases among Young children in low-income countries (BIRDY) program, a surveillance program of bacterial infections among young children in low- and middle-income countries (*6*). During September 2015–December 2016, ESBL-producing *E. coli* isolates were cultured from rectal swabs or fecal samples collected at or just after delivery (Appendix Table 3).

We further included ESBL-producing *E. coli* from 15 Phnom Penh–based patients who sought care at the Sihanouk Hospital Center of Hope during November 2015–December 2016. ESBL-producing *E. coli* were cultured from blood (12 patients), urine (2 patients), and peritoneal fluid (1 patient) (Appendix Table 4).

**Figure 1:**
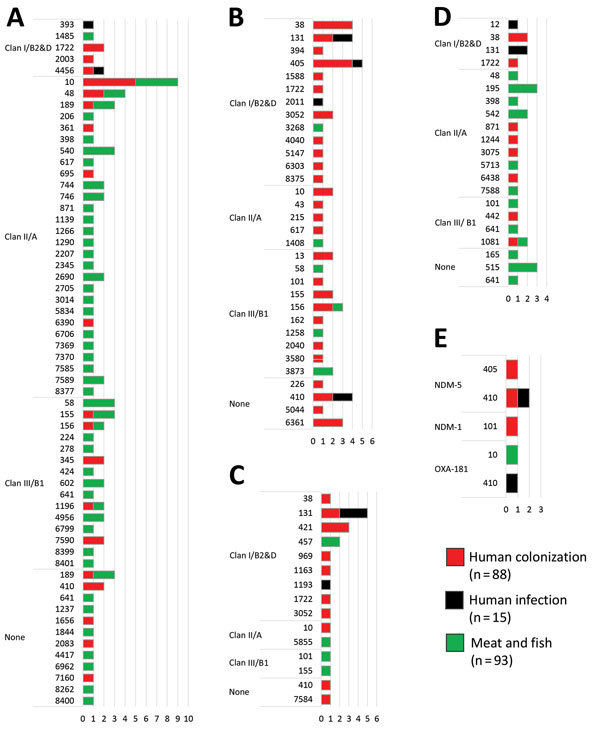
Distribution of 105 multilocus sequence types (MLSTs) among predominant extended-spectrum β-lactamase (ESBL) and carbapenemase gene types encoded by 196 ESBL-producing *Escherichia coli* from humans and food, Cambodia, 2015–2016. A) CTX-M-55; B) CTX-M15; C) CTX-M-27; D) CTX-M-14; E) carbapenemases. Vertical axes depict MLSTs. Horizontal axes depict the frequency of each observed MLST. CTX-M-3, CTX-M-24, and CTX-M-65 are not shown because these ESBL gene types were rare (<2%). One human colonization isolate (ST394, clan I/B2&D) encoded CTX-M-3, 1 food-origin isolate (ST10, clan II/A) encoded CTX-M-24, and 2 food-origin isolates (ST2207, clan II/A and ST7586, clan III/B1) encoded CTX-M-65.

We performed whole-genome sequencing for 1 ESBL-producing *E. coli* isolate from each food sample and all human-origin ESBL-producing *E. coli* isolates (Appendix Sections 1.4–1.6) and compiled genetic and phenotypic characteristics of these196 isolates (Appendix Tables 6, 7). We also determined distribution of multilocus sequence types (MLSTs) encoding predominant ESBL- or carbapenemase-gene types (Figure 1). 

**Figure 2:**
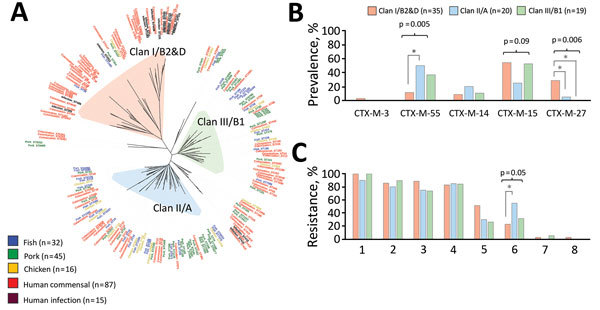
Genomic comparisons of extended-spectrum β-lactamase (ESBL)–producing *Escherichia coli* from humans, fish, pork, and chicken from Cambodia and differences in human colonization isolates by phylogenetic clan. All isolates were phenotypically resistant to third-generation cephalosporins (data not shown). A) Whole-genome sequence-based phylogenetic tree of 195 ESBL-producing *E. coli* genomes comprising 87 human colonization isolates, 15 human clinical isolates, and 93 isolates from fish, pork, and chicken meat and resulting phylogenetic clans I/B2&D (n = 53), II/A (n = 69), and III/B1 (n = 47). B) ESBL-encoding genes of human colonization *E. coli* isolates, by phylogenetic clan. C) Phenotypic resistance of human colonization ESBL-producing *E. coli* isolates to antimicrobial drugs of 8 classes, by phylogenetic clan. Clinical isolates are not included in panels B or C. Of 87 human colonization genomes, 13 did not group into a phylogenetic clan and thus are excluded from panels B and C. Prevalence of outcome differed significantly (p<0.05, indicated by *) between 2 indicated clans by post hoc Tukey test. Only statistically significant differences are depicted. 1, quinolone; 2, co-trimoxazole; 3, tetracycline; 4, aminoglycoside; 5, macrolide; 6, amphenicol; 7, carbapenem; 8, colistin.

Phylogenetic analysis of ESBL-producing *E. coli* genomes revealed 3 distinct clans (Figure 2, panel A). Clan I/B2&D (n = 53) comprised mostly human-origin isolates, including isolates from colonized persons and most infected patients. Clans II/A (n = 69) and III/B1 (n = 47) included isolates from colonized persons and from food but not from infected patients. Each clan comprised an exclusive subset of sequence types (STs); clan I/B2&D included ST131 and clonal complex (CC) 38, clan II/A included CC10, and clan III/B1 included CC58 and CC156. Approximately half (21/39) of isolates in clans II/A and III/B1 from colonized patients belonged to STs detected in both humans and meat (Appendix Table 8).

We determined the distributions of ESBL-encoding genes and resistance patterns among isolates from colonized persons by clan (Figure 2, panels B and C). The *bla*_CTX-M-55_ gene was more common among colonization isolates belonging to clan II/A than to clan I/B2&D (p<0.05). Amphenicol resistance was more common among colonization isolates belonging to clan II/A than clan I/B2&D (p<0.05) and was most often encoded by *flo*R (Appendix Table 7).

Women colonized with amphenicol-resistant (vs. amphenicol-susceptible) ESBL-producing *E. coli* were more likely to report having ever eaten dried poultry (adjusted odds ratio 9.0, 95% CI 1.8–45.2) (Table). Women colonized with CTX-M-55–producing *E. coli* (vs. other ESBL types) were more likely to have handled live poultry (adjusted odds ratio 4.6, 95% CI 1.1–19.3), but this exposure was uncommon (11/88).

**Table:** Environmental exposures and colonization with chloramphenicol-resistant and CTX-M-55–encoding ESBL-producing *Escherichia coli* among healthy women*,* Phnom Penh, Cambodia, 2015–2016

Variable	CHL resistance					ESBL type			
Resistant, no. (%), n = 29	Susceptible, no. (%), n = 59	OR (95% CI)	aOR (95% CI)	CTX-M-55, no. (%), n = 26	Other, no. (%), n = 62	OR (95% CI)	aOR (95% CI)
Persons living in home									
>8	5 (17)	10 (17)	1.1 (0.3–3.7)			3 (12)	12 (19)	0.6 (0.1–2.5)	
6–8	9 (31)	19 (32)	1.1 (0.3–3.7)			10 (38)	18 (29)	1.4 (0.5–3.7)	
<5	15 (52)	30 (51)	Referent			13 (50)	32 (52)	Referent	
Place of delivery									
Private clinic	5 (17)	17 (29)	0.4 (0.1–1.4)			4 (15)	18 (29)	0.4 (0.1–1.4)	
Hospital	11 (38)	20 (34)	0.8 (0.3–2.2)			9 (35)	22 (35)	0.7 (0.2–1.9)	
Health center	13 (45)	22 (37)	Referent			13 (50)	22 (35)	Referent	
Received antimicrobial drugs at delivery†	2 (7)	11 (19)	0.3 (0.1–1.3)	0.2 (0.0–1.1)		1 (4)	12 (19)	0.2 (0–1.3)	0.2 (0.0–1.4)
Untreated drinking water	5 (17)	7 (12)	1.5 (0.4–5.3)			4 (15)	8 (13)	1.2 (0.3–4.5)	
Toilet shared‡	11 (38)	16 (27)	1.6 (0.6–4.2)			5 (19)	22 (35)	0.4 (0.1–1.3)	
Nonflush toilet	26 (90)	47 (80)	2.2 (0.6–8.5)			24 (92)	49 (79)	3.2 (0.7–15.3)	
Pet contact	6 (21)	13 (22)	0.9 (0.3–2.7)			6 (23)	13 (21)	1.1 (0.4–3.4)	
Live poultry contact	4 (14)	7 (12)	1.2 (0.3–4.4)			6 (23)	5 (8)	3.4 (0.9–12.4)	4.6 (1.1–19.3)
Consumption habits									
Dried pork >1×/wk	15 (52)	32 (54)	0.9 (0.4–2.2)			11 (42)	36 (58)	0.5 (0.2–1.3)	
Dried beef	17 (59)	38 (64)	0.8 (0.3–2.1)			20 (77)	35 (56)	2.6 (0.9–7.3)	
Dried poultry	27 (93)	39 (66)	7.9 (1.7–36.4)	9.0 (1.8–45.2)		22 (85)	44 (71)	2.3 (0.7–7.5)	
Pork >3×/wk	22 (76)	53 (90)	0.4 (0.1–1.2)	0.2 (0.1–1.1)		23 (88)	52 (84)	1.5 (0.4–5.9)	
Insects	21 (72)	33 (56)	2.2 (0.8–5.7)			16 (62)	38 (61)	1 (0.4–2.6)	
Raw vegetables >1×/wk	5 (17)	8 (14)	1.3 (0.4–4.5)			3 (12)	10 (16)	0.7 (0.2–2.7)	

*Blank cells indicate variable not included in multivariate models. aOR, adjusted (for age) OR; CHL, chloramphenicol; ESBL, extended-spectrum β-lactamase; OR, odds ratio.  †Not reported for 4 women (missing data). All 4 were colonized with CHL-susceptible ESBL-producing *Escherichia coli*. One woman was colonized with CTX-M-55–type *E. coli*, whereas the other 3 were colonized with other CTX-M–encoded isolates. ‡With persons in other households.

Our genomic and epidemiologic findings suggest that ESBL-producing *E. coli* that contaminates meat and fish in Phnom Penh may be disseminating to the community. ESBL-producing *E. coli* were highly prevalent among the meat and fish we sampled. More than 80% of food-origin isolates were amphenicol resistant, and two thirds produced CTX-M-55. When food-origin isolates were compared with human-origin isolates, ≈40% of ESBL-producing *E.coli* from healthy persons grouped into the same phylogenetic clans that comprised most food-origin isolates. Approximately half of these colonization isolates had MLSTs detected among food, and a substantial portion were more likely to produce CTX-M-55 and be amphenicol resistant than colonization isolates that grouped separately. The fact that chloramphenicol has not been used in human medicine for almost 20 years in Cambodia, yet chloramphenicol analogs (e.g., florfenicol, thiamphenicol) are administered to food animals (*5*,*7*), suggests a food origin for these colonizing isolates.

Healthy women colonized with amphenicol-resistant ESBL-producing *E. coli* were more likely to eat poultry meat prepared by sun drying, a process that may not eliminate bacteria (*8*). Although we did not test dried meat samples for ESBL-producing *E. coli* contamination, our finding is consistent with those of other studies (*8*,*9*). Women reported having prepared dried poultry at home. Especially in low-resource households, sun-dried meat may become cross-contaminated by raw meat, dust, animals, and flies (*8*).

Our findings are concerning because of growing interest in using chloramphenicol as a drug of last resort for panresistant strains of bacteria (*10*). In the early 2000s, the Cambodia government stopped purchasing chloramphenicol because of concerns about side effects. Since restriction of this drug, infections in the hospital setting have reverted to a chloramphenicol-susceptible phenotype (*11*). Nevertheless, our findings suggest that amphenicol resistance genes are circulating in the community, potentially because amphenicol use in food animals has selected for resistant bacteria that can spread to humans (*12*). This possibility is concerning because physicians in Cambodia are often unable to assess the resistance of infectious agents before prescribing antimicrobial drugs (*4*).

Our study had several limitations. First, for logistical reasons, we sampled meat and fish during only 1 season. Contamination rates may have differed had we sampled across seasons (*13*). Second, although we included colonization samples from healthy women, all women had recently given birth in healthcare settings. However, more than half were colonized with ESBL-producing *E. coli* phylo-types A and B1, supporting community-associated, rather than healthcare-associated, acquisition. Third, we were unable to include clinical isolates from the same population that contributed colonization isolates. Thus, differences in colonization and clinical isolates could have resulted from population differences. Fourth, we did not sample food animals, which could have helped confirm that CTX-M-55–type and amphenicol-resistant ESBL-producing *E. coli* circulate among them. Last, we did not investigate additional potential pathways for ESBL-producing *E. coli* transmission to colonized women, such as contact with persons employed at farms or slaughterhouses or proximity to such operations.

## Conclusions

This study, which integrated epidemiologic and genomic methods to characterize community, clinical, and environmental data, supports concerns that the dissemination of antimicrobial drug–resistant bacteria from food animals to humans may be more likely in low- and middle-income countries (*14*,*15*). This finding is concerning because meat consumption is projected to drastically increase in these countries, and animal production that relies on routine antimicrobial drug use is being promoted to meet this demand (*14*). Particularly for low- and middle-income countries such as Cambodia, implementation of multisectoral strategies to combat antimicrobial resistance from a One Health perspective must be supported, and food safety should be prioritized.

Appendix Additional methods and results from study of meat and fish as sources of extended-spectrum β-lactamase–producing *Escherichia coli*, Cambodia.
