# Supplementary material for: Meat and Fish as Sources of Extended-Spectrum β-Lactamase–Producing Escherichia coli, Cambodia
Source: Emerg Infect Dis. 2019 Jan;25(1):126–31. doi: 10.3201/eid2501.180534 (PMC6302604; doi:10.3201/eid2501.180534)
Supplement: Appendix — Additional methods and results from study of meat and fish as sources of extended-spectrum β-lactamase–producing Escherichia coli, Cambodia. [file 18-0534-Techapp-s1.pdf]

Article DOI: https://doi.org/10.3201/eid2501.180534

Meat and Fish as Sources of Extended-Spectrum β-lactamase–Producing *Escherichia coli*, Cambodia

Appendix

1. Supplementary Methods

1.1. Detection of third generation cephalosporin- and carbapenem-resistant *Escherichia coli* from fish and meat

All samples were processed within two hours of arrival at IPC. First, 10 g of sample were homogenized in 90 ml Brain Heart Infusion Broth (BHIB). For pork and fish, we sub-sampled meat from both the surface and interior. For chicken, we sub-sampled neck skins only, as is typical when sampling whole chicken carcasses (*1*). Following overnight incubation at 37°C, a sterile loop was used to plate ~10 µl of enriched BHIB onto Drigaliski supplemented with 2 mg/L cefotaxime (DRI-CTX), to select for third-generation cephalosporin -resistant *Enterobactericeae*, and Drigaliski supplemented with 0.5 mg/L ertapenem (DRI-ERT), to select for carbapenemase-producing *Enterobacteriaceae*. Plates were incubated overnight at 37°C. We subcultured up to two lactose-producing colonies from DRI-CTX and DRI-ERT for further characterization.

To confirm ESBL production among presumptive *E. coli* selected from DRI-CTX, we performed the double-disk synergy test with aztreonam (monobactam), cefotaxime, ceftazidime (third generation cephalosporins), cefepime (fourth generation cephalosporin), and an amoxicillin-clavulanate disc. Isolates for which we observed an enhanced inhibition zone toward amoxicillin-clavulanate were considered ESBL-producers.

To confirm carbapenemase production among presumptive *E. coli* selected from DRI-ERT, we performed the Carba-NP test (*2*). Isolates that produced a color change within two hours were considered carbapenemase-producers.

1.2. Species identification

Among ESBL-P and carbapenemase-producing isolates, we used API20E to confirm the species of up to one presumptive *E. coli* per sample.

1.3. Antibiotic resistance testing

One third-generation cephalosporin- and/or carbapenem-resistant *E. coli* isolate per food sample was assessed for resistance to nine antibiotics at IPC, using the Kirby-Bauer disk diffusion method. All human-origin ESBL-*Ec* were assessed for resistance to 30 antibiotics at IP-Paris (Appendix, Table 1). Diameter interpretations were based on 2016 European Committee on Antimicrobial Susceptibility Testing (EUCAST) recommendations where available, or 2016 Clinical and Laboratory Standards Institute (CLSI) recommendations for those antibiotics for which 2016 EUCAST recommendations did not exist (*3*,*4*). MICs (MICs) for azithromycin, nalidixic acid, and ciprofloxacin were additionally determined used E-tests (bioMérieux, France). We defined isolates resistant to ≥3 antibiotic classes (including third-generation cephalosporins) as multidrug-resistant.

We screened all isolates for colistin susceptibility using a 4 mg/L colistin sulfate solution. Among strains that exhibited growth following overnight incubation at 37°C, we used Sensititer colistin microdilution assays (TREK Diagnostic Systems Inc., Cincinnati, OH) to determine colistin MICs.

1.4. Genome characterization

Libraries were constructed using the Nextera XT DNA Library Preparation kit (Illumina, Inc., San Diego, CA) and sequenced on a NextSeq-500 instrument using a 2x150 paired-end protocol. All sequenced paired-ends reads were clipped and trimmed with AlienTrimmer (*5*), corrected with Musket (*6*), merged (if needed) with FLASH (*7*), and subjected to a digital normalization procedure with khmer (*8*). For each sample, remaining processed reads were assembled and scaffolded with SPAdes (*9*).

E. coli genomes were screened for acquired antimicrobial resistance genes with ResFinder (selected threshold equal to 90% identity), assigned a multilocus-sequence type (MLST) based on the Achtman scheme (*10,11*), and assigned a core-genome MLST (cgMLST) based on a scheme from Enterobase that uses 2,513 loci. *E. coli* clonal complexes were

determined using goeBURST following the stringent group definition (6/7 shared alleles) (*12*). We used in-silico PCR to assign phylo-types following the Clermont scheme (13).

Sequence data have been deposited in the European Nucleotide Archive (http://www.ebi.ac.uk/ena) under project number PRJEB25898 (Appendix Table 5).

1.5. Phylogenetic analysis

A Minimum Evolution phylogenetic tree was inferred from the pairwise evolutionary distances estimated between each pair of assembled ESBL-*Ec* genomes. We used this approach because our goal was to examine the general population structure of epidemiologically-unrelated isolates belonging to 100 STs (with no more than 13 isolates belonging to any single ST (Appendix Table 8)), rather to investigate molecular evidence for specific cases of transmission.

The pairwise *p*-distance (i.e., proportion of nucleotide differences) between each pair of whole genome sequences was estimated with Mash (*14*). To infer accurate *p*-distances, *k*-mer size = 20 was chosen according to the Mash method recommendation (see Ondov *et al*, formula (*2*)), and sketch size = 431,000 was selected by searching for the one that leads to the pairwise distance matrix associated with the optimal overall treelikeness (*15*). As every pairwise *p*-distance was quite small (i.e., all Mash estimates <0.038), no further correction was required (*16,17*), and the distance matrix was directly used for a distance-based phylogenetic inference with FastMe (*18*). One human colonization ESBL-*Ec* was excluded from genomic comparisons due to insufficient quality.

The resulting tree was visualized using iTOL v4.2 (https://itol.embl.de/) (*19*). We use the term “clan” rather than “clade” when reporting results to clarify our description of an unrooted tree (*20*).

To ensure that the inclusion of accessory genomes in our whole genome-based phylogenetic analysis did not bias our results, we performed a phylogenetic analysis of the 2,513 cgMLST loci defined by the Enterobase scheme. For each locus, allele sequences were aligned with MAFFT (*21*), and a Maximum Likelihood phylogenetic tree was inferred using IQTree (optimal evolutionary model GTR+F+R3) from the concatenation of the 2,513 multiple sequence alignments (Appendix Figure). The “clans” delineated by this cgMLST-based phylogenetic tree were nearly identical to those presented in Figure 2, suggesting that our whole genome-based

phylogenetic approach did not significantly alter the overall, population-level relationships that we were interested in characterizing.

1.6. Statistical analyses

We used Fisher exact tests to compare resistance patterns among ESBL-*Ec* from different meat types and between human sample types (*i.e.*, colonization, infection).

Among human colonization ESBL-*Ec*, we examined whether ESBL-encoding genes and phenotypic antibiotic resistance patterns differed between phylogenetic clans using one-way ANOVAs and post-hoc Tukey tests. Both a) amphenicol resistance and b) presence of $bla_{\text{CTX-M-55}}$ significantly differed between clans ($p < 0.05$ by Tukey test). Thus, we constructed univariate logistic regression models examining associations between healthy women's environmental exposures, including dietary habits, and the presence versus absence of these characteristics in their colonizing ESBL-*Ec* (i.e., amphenicol resistance versus susceptibility, CTX-M-55 versus other ESBL-type). Additionally, we used multinomial logistic regression models to explore associations between healthy women's exposures and the phylogenetic clan (*i.e.*, I/B2&D, II/A, or III/B1) to which their colonizing ESBL-*Ec* belonged. Exposures considered for both model types are listed in Appendix Table 3.

Variables with univariate *p*-values ≤0.2 were included in multivariate binary and multinomial logistic regression models, respectively. We conducted backward stepwise elimination of non-significant parameters ($p < 0.05$). Multivariate models were adjusted for age.

Genomic differences were not explored for clinical ESBL-*Ec* because most clinical isolates grouped in one phylogenetic clan.

Analyses were performed using SAS version 9.4 (Cary, NC).

2. Supplementary Results

2.1. Characteristics of ESBL-*Ec* from food and humans

ESBL genes. Among 93 ESBL-*Ec* from food, CTX-M-55 was the most common ESBL gene type detected, comprising 23/32 ESBL-*Ec* from fish (72%), 27/45 from pork (60%), and 12/16 (75%) from chicken (Appendix Table 6).

Among 88 human colonization isolates, CTX-M-15 (41/88) and CTX-M-55 (27/88) were the most common ESBL gene types, while among 15 clinical isolates, CTX-M-15 (6/15) was most common and CTX-M-55 (2/15) was least common (Appendix Table 7).

Antibiotic resistance. More than two-thirds of ESBL-*Ec* from food (62/93) expressed resistance to at least five antibiotic classes in addition to third-generation cephalosporins, most commonly tetracycline (89%), co-trimoxazole (86%), fluoroquinolone (80%), aminoglycoside (86%), and amphenicol (83%). We identified 11 phenotypically colistin-resistant ESBL-*Ec* from three fish and eight pork, but none from chicken. Colistin resistance among 3/3 isolates from fish and 4/8 from pork was mediated solely by *mcr*-1, resistance among 1/8 pork was mediated solely by *mcr*-3, and resistance among 2/8 pork was mediated by both *mcr*-1 and *mcr*-3 (Appendix Table 6).

Human colonization isolates were more likely to be resistant to amphenicol ($p = 0.06$) and susceptible to carbapenems ($p = 0.04$) and azithromycin ($p = 0.02$) than clinical isolates. Carbapenem resistance was mainly encoded by NDM-type genes. Colistin resistance was rare (<3%) among colonization isolates and was not detected among clinical isolates (Table 4).

MLST. We detected 105 distinct STs. Ten of these 105 STs (10%) were detected among both humans and food (i.e., STs 10, 48, 101, 155, 156, 189, 617, 871, 1081, 1196), while 44/105 and 51/105 were detected exclusively among humans or food, respectively (Appendix Table 8). ST10 and single locus variants (collectively, clonal complex (CC) 10) were the most common STs among both food-origin and human colonization ESBL-*Ec*, comprising 11/93 isolates (12%) and 12/88 isolates, respectively (14%). STs 131 and 410 were more common among clinical isolates, comprising 7/15 (47%) and 2/15 (13%) isolates, respectively.

CC10 encoded all predominant ESBL gene types, although CTX-M-55 was most common (Figure 1; Appendix Table 8). STs that were only common among human-origin isolates (*i.e.* ST131, CC38, ST410, ST405) rarely or never encoded $bla_{\text{CTX-M-55}}$. Instead, ST131 (n = 11) mostly encoded $bla_{\text{CTX-M-15}}$ (4/11) and $bla_{\text{CTX-M-27}}$ (5/11), CC38 (n = 11) mostly encoded $bla_{\text{CTX-M-15}}$ (7/11), ST410 (n = 6) mostly encoded $bla_{\text{CTX-M-15}}$ (4/6), and ST405 (n = 5) exclusively encoded $bla_{\text{CTX-M-15}}$.

Although only 10/105 (10%) STs were shared between humans and food, 22/88 (25%) of human colonization isolates belonged to these STs. Among colonization isolates that grouped in Clans II/A and III/B1, 21/39 (54%) belonged to nine shared STs.

2.2. Environmental exposures associated with humans' ESBL-*Ec* colonization patterns

We did not identify consistent associations between any of the environmental or healthcare exposures we examined and women's colonization with ESBL-*Ec* that belonged to clans II/A or III/B1, versus I/B2&D (referent) (Appendix Table 9).

3. Supplementary Discussion

Our findings differ from previous studies conducted in Europe. Although ESBL-*Ec* are prevalent among poultry (≥80%) in several European countries (*22*), they are usually genetically distinct from ESBL-*Ec* circulating among healthy humans (*23*). Conversely, we report a substantial portion of isolates from healthy, gut-colonized persons that were phylogenetically related to food-origin strains. Similar to European studies, we found that only 10% of MLSTs were shared between human- and food-origin ESBL-*Ec*. However, 25% of human colonization isolates belonged to these overlapping STs, and this proportion was even higher (54%) among colonization isolates that were phylogenetically related to food-origin ESBL-*Ec*. In comparison to Europe, we conjecture that weaker public health protections, inadequate regulation of antibiotic use in food animals, and/or consumption of undercooked animal products could be exacerbating the spread of bacterial clones and ESBL-encoding mobile genetic elements from farmed animals to the community in Phnom Penh.

CTX-M-55 is an increasingly reported ESBL gene type among humans, farmed animals, food, and the environment in Asia (*24–26*), and was the most common ESBL gene type recovered from fish and meat in this study. We were unable to identify dietary exposures that were associated with women's colonization with CTX-M-55-producing *E. coli*, although women colonized with these isolates were more likely to report direct contact with live poultry. Other work suggests this ESBL-type may be widespread in the environment (*25*), and thus tracing community exposure pathways may have been difficult.

Among the samples we tested, pork was most commonly contaminated with ESBL-*Ec* (75%). This finding was unexpected because other studies have found poultry and poultry meat

to be most frequently contaminated (*22,27*). In Europe, ESBL selection is thought to be a consequence of third-generation cephalosporin administration to eggs and young chicks (*28*), but in Cambodia, farming practices that might select for ESBLs are not monitored (*29*). Our finding that pork was more contaminated could be a reflection of higher prevalence of ESBL-*Ec* fecal carriage among pigs compared to chickens, as has been observed in Thailand (*30*), or the fact that pork is more heavily processed than chicken or fish before sale. Future studies should include samples from the food supply chain to investigate sources of contamination.

Unlike colonization isolates, none of the clinical isolates we examined grouped in the phylogenetic clans that comprised most food-origin isolates (Clans II/A and III/B1). One hypothesis for this finding is that ESBL-*Ec* with characteristics of food animal origin are less capable of causing infections. However, we lacked sufficient diversity in our clinical isolates to investigate this possibility. Specifically, as gut-colonizing *E. coli* are more likely to cause urinary tract infections (UTIs) than systemic infections, this hypothesis would have been best explored with the inclusion of a much larger number of UTIs. However, UTIs are difficult to sample in Cambodia and other LMICs where antibiotics can be purchased without a prescription, as sick persons rarely seek medical care for uncomplicated cases. Thus, we were only able to include ESBL-*Ec* from two UTIs in our present analysis. Future studies in LMICs should prioritize inclusion of UTI samples to fully investigate this hypothesis.

Although all ESBL-*Ec* from colonized humans that grouped in Clan I were phylo-types B2 or D (commonly associated with infection), we did not identify healthcare exposures associated with women's colonization with these isolates. Global studies have described a high proportion of ESBL-*Ec* belonging to phylo-types B2&D, including ST131, among gut-colonized, healthy individuals who lack recent healthcare exposures (*31*). Indeed, the fact that most clinical isolates that grouped in Clan I/B2&D were community-associated (12/13), rather than hospital-associated, suggests that these phylo-types and STs may be circulating in the community. However, we did observe that women who received antibiotics during delivery were less likely to carry ESBL-*Ec* with genetic and phenotypic characteristics of food-origin isolates (*i.e.* CTX-M-55 and amphenicol resistance), although neither of these results were statistically significant. It is possible that antibiotic exposure during delivery altered these women's intestinal flora, facilitating colonization with ESBL-*Ec* that encoded different CTX-M-types and resistance patterns than those which predominated among food-origin isolates.

ESBL-*Ec* we detected on meat and fish could have originated from human contamination, including from farmers (if animals were exposed to human waste), or by slaughterhouse workers and market vendors, through handling. However, >80% of ESBL-*Ec* from meat and fish were resistant to amphenicols, an antibiotic class that has not been used by humans in Cambodia for almost 20 years. If the meat and fish we sampled were primarily contaminated with human-origin ESBL-*Ec*, we would have expected a much smaller proportion of these isolates to be amphenicol-resistant (perhaps similar to what we found among colonized women, for example, *i.e.* 33%). This discrepancy suggests that food animals, who are regularly given amphenicols (*32*), were the main source of the ESBL-*Ec* strains we recovered from animal-derived products.

References

1. Cox NA, Richardson LJ, Cason JA, Buhr RJ, Vizzier-Thaxton Y, Smith DP, et al. Comparison of neck skin excision and whole carcass rinse sampling methods for microbiological evaluation of broiler carcasses before and after immersion chilling. J Food Prot. 2010;73:976–80. PubMed http://dx.doi.org/10.4315/0362-028X-73.5.976

2. Nordmann P, Poirel L, Dortet L. Rapid detection of carbapenemase-producing Enterobacteriaceae. Emerg Infect Dis. 2012;18:1503–7. PubMed http://dx.doi.org/10.3201/eid1809.120355

3. Clinical and Laboratory Standards Institute (CLSI). Performance Standards for Antimicrobial Susceptiblity Testing—26th ed. (M100S). Wayne (PA): The Institute; 2016.

4. European Committee on Antimicrobial Susceptibility Testing (EUCAST). Breakpoint tables for interpretation of MICs and zone diameters, Version 6.0. 2016 [cited 2018 Nov 1]. http://www.eucast.org/fileadmin/src/media/PDFs/EUCAST_files/Breakpoint_tables/v_6.0_Breakpoint_table.pdf

5. Criscuolo A, Brisse S. AlienTrimmer: a tool to quickly and accurately trim off multiple short contaminant sequences from high-throughput sequencing reads. Genomics. 2013;102:500–6. PubMed http://dx.doi.org/10.1016/j.ygeno.2013.07.011

6. Liu Y, Schröder J, Schmidt B. Musket: a multistage k-mer spectrum-based error corrector for Illumina sequence data. Bioinformatics. 2013;29:308–15. PubMed http://dx.doi.org/10.1093/bioinformatics/bts690

7. Magoč T, Salzberg SL. FLASH: fast length adjustment of short reads to improve genome assemblies. Bioinformatics. 2011;27:2957–63. PubMed http://dx.doi.org/10.1093/bioinformatics/btr507

8. Crusoe MR, Alameldin HF, Awad S, Boucher E, Caldwell A, Cartwright R, et al. The khmer software package: enabling efficient nucleotide sequence analysis. F1000Res. 2015;4:900. PubMed

9. Bankevich A, Nurk S, Antipov D, Gurevich AA, Dvorkin M, Kulikov AS, et al. SPAdes: a new genome assembly algorithm and its applications to single-cell sequencing. J Comput Biol. 2012;19:455–77. PubMed http://dx.doi.org/10.1089/cmb.2012.0021

10. Zankari E, Hasman H, Cosentino S, Vestergaard M, Rasmussen S, Lund O, et al. Identification of acquired antimicrobial resistance genes. J Antimicrob Chemother. 2012;67:2640–4. PubMed http://dx.doi.org/10.1093/jac/dks261

11. Larsen MV, Cosentino S, Rasmussen S, Friis C, Hasman H, Marvig RL, et al. Multilocus sequence typing of total-genome-sequenced bacteria. J Clin Microbiol. 2012;50:1355–61. PubMed http://dx.doi.org/10.1128/JCM.06094-11

12. Francisco AP, Bugalho M, Ramirez M, Carriço JA. Global optimal eBURST analysis of multilocus typing data using a graphic matroid approach. BMC Bioinformatics. 2009;10:152. PubMed http://dx.doi.org/10.1186/1471-2105-10-152

13. Clermont O, Christenson JK, Denamur E, Gordon DM. The Clermont *Escherichia coli* phylo-typing method revisited: improvement of specificity and detection of new phylo-groups. Environ Microbiol Rep. 2013;5:58–65. PubMed http://dx.doi.org/10.1111/1758-2229.12019

14. Ondov BD, Treangen TJ, Melsted P, Mallonee AB, Bergman NH, Koren S, et al. Mash: fast genome and metagenome distance estimation using MinHash. Genome Biol. 2016;17:132. PubMed http://dx.doi.org/10.1186/s13059-016-0997-x

15. Holland BR, Huber KT, Dress A, Moulton V. δ plots: a tool for analyzing phylogenetic distance data. Mol Biol Evol. 2002;19:2051–9. PubMed http://dx.doi.org/10.1093/oxfordjournals.molbev.a004030

16. Nei M, Kumar S. Molecular Evolution and Phylogenetics. New York: Oxford University Press; 2000.

17. Nei M, Zhang J. Evolutionary Distance: Estimation. In: eLS. John Wiley & Sons, Ltd; 2001. http://dx.doi.org/10.1038/npg.els.0005108

18. Lefort V, Desper R, Gascuel O. FastME 2.0: A Comprehensive, Accurate, and Fast Distance-Based Phylogeny Inference Program. Mol Biol Evol. 2015;32:2798–800. PubMed http://dx.doi.org/10.1093/molbev/msv150

19. Letunic I, Bork P. Interactive tree of life (iTOL) v3: an online tool for the display and annotation of phylogenetic and other trees. Nucleic Acids Res. 2016;44(W1):W242-5. PubMed http://dx.doi.org/10.1093/nar/gkw290

20. Wilkinson M, McInerney JO, Hirt RP, Foster PG, Embley TM. Of clades and clans: terms for phylogenetic relationships in unrooted trees. Trends Ecol Evol. 2007;22:114–5. PubMed http://dx.doi.org/10.1016/j.tree.2007.01.002

21. Katoh K, Misawa K, Kuma K, Miyata T. MAFFT: a novel method for rapid multiple sequence alignment based on fast Fourier transform. Nucleic Acids Res. 2002;30:3059–66. PubMed http://dx.doi.org/10.1093/nar/gkf436

22. Lazarus B, Paterson DL, Mollinger JL, Rogers BA. Do human extraintestinal Escherichia coli infections resistant to expanded-spectrum cephalosporins originate from food-producing animals? A systematic review. Clin Infect Dis. 2015;60:439–52. PubMed http://dx.doi.org/10.1093/cid/ciu785

23. Dorado-García A, Smid JH, van Pelt W, Bonten MJM, Fluit AC, van den Bunt G, et al. Molecular relatedness of ESBL/AmpC-producing Escherichia coli from humans, animals, food and the environment: a pooled analysis. J Antimicrob Chemother. 2017. In press. PubMed

24. Zhang J, Zheng B, Zhao L, Wei Z, Ji J, Li L, et al. Nationwide high prevalence of CTX-M and an increase of CTX-M-55 in Escherichia coli isolated from patients with community-onset infections in Chinese county hospitals. BMC Infect Dis. 2014;14:659. PubMed http://dx.doi.org/10.1186/s12879-014-0659-0

25. Runcharoen C, Raven KE, Reuter S, Kallonen T, Paksanont S, Thammachote J, et al. Whole genome sequencing of ESBL-producing Escherichia coli isolated from patients, farm waste and canals in Thailand. Genome Med. 2017;9:81. PubMed http://dx.doi.org/10.1186/s13073-017-0471-8

26. Zheng H, Zeng Z, Chen S, Liu Y, Yao Q, Deng Y, et al. Prevalence and characterisation of CTX-M β-lactamases amongst Escherichia coli isolates from healthy food animals in China. Int J Antimicrob Agents. 2012;39:305–10. PubMed http://dx.doi.org/10.1016/j.ijantimicag.2011.12.001

27. Overdevest I, Willemsen I, Rijnsburger M, Eustace A, Xu L, Hawkey P, et al. Extended-spectrum β-lactamase genes of Escherichia coli in chicken meat and humans, The Netherlands. Emerg Infect Dis. 2011;17:1216–22. PubMed http://dx.doi.org/10.3201/eid1707.110209

28. Baron S, Jouy E, Larvor E, Eono F, Bougeard S, Kempf I. Impact of third-generation-cephalosporin administration in hatcheries on fecal Escherichia coli antimicrobial resistance in broilers and layers. Antimicrob Agents Chemother. 2014;58:5428–34. PubMed http://dx.doi.org/10.1128/AAC.03106-14

29. Om C, McLaws M-L. Antibiotics: practice and opinions of Cambodian commercial farmers, animal feed retailers and veterinarians. Antimicrob Resist Infect Control. 2016;5:42. PubMed http://dx.doi.org/10.1186/s13756-016-0147-y

30. Boonyasiri A, Tangkoskul T, Seenama C, Saiyarin J, Tiengrim S, Thamlikitkul V. Prevalence of antibiotic resistant bacteria in healthy adults, foods, food animals, and the environment in selected areas in Thailand. Pathog Glob Health. 2014;108:235–45. PubMed http://dx.doi.org/10.1179/2047773214Y.0000000148

31. Nicolas-Chanoine M-H, Gruson C, Bialek-Davenet S, Bertrand X, Thomas-Jean F, Bert F, et al. 10-Fold increase (2006-11) in the rate of healthy subjects with extended-spectrum β-lactamase-producing Escherichia coli faecal carriage in a Parisian check-up centre. J Antimicrob Chemother. 2013;68:562–8. PubMed http://dx.doi.org/10.1093/jac/dks429

32. Ström G, Boqvist S, Albihn A, Fernström L-L, Andersson Djurfeldt A, Sokerya S, et al. Antimicrobials in small-scale urban pig farming in a lower middle-income country - arbitrary use and high resistance levels. Antimicrob Resist Infect Control. 2018;7:35. PubMed http://dx.doi.org/10.1186/s13756-018-0328-y

4. Supplementary Tables

Appendix Table 1. Antibiotics used for susceptibility testing of human- and food-origin *Escherichia coli* isolates.

Antibiotic class	Antibiotic tested	Laboratory		Disk Concentration (μg)	Zone Diameter Thresholds[a] (mm)	MIC (MIC) Thresholds[b] (μg/mL)
		IPC	IP-Paris			
Amphenicol	Chloramphenicol		X	30	17–17	
Β-lactam	Amoxacillin	X		25	14–14	
	Ampicillin		X	10	14–14	
	Ticarcillin		X	75	23–23	
	Piperacillin		X	30	17–20	
Β-lactam+ Β-lactamase inhibitor	Piperacilin+tazobactum	X	X	30/6	17–20	
	Amoxacillin+clavulanic acid[c]	X	X	20/10	19–19	
	Ticarcillin+ clavulanic acid		X	75/10	23–23	
Carbapenem	Ertapenem	X	X	10	22–25	
	Imipenem	X	X	10	16–22	
	Meropenem		X	10	16–22	
Cephalosporin	Cefepime[c]	X	X	30	21–24	
	Ceftazidime[c]	X	X	10	19–22	
	Cefamandole[d]		X	30	15–18	
	Cefapezarone[d]		X	30	16–21	
	Cefoxitin		X	30	19–19	
	Cefoxatime[c]	X	X	5	17–20	
Aminoglycoside	Gentamicin	X	X	10	14–17	
	Streptomycin[d]		X	10	12–15	
	Kanamycin[d]		X	30	14–18	
	Netilimicine		X	10	12–15	
	Amikacine		X	30	15–18	
Fluoroquinolone	Ciprofloxacin	X	X	5	19–22	0.5–1
	Nalidixic acid[d]	X	X	30	14–19	16–32
	Perfloxacin		X	5	24–24	
Macrolide	Azithromycin[d]		X	15	13–13	16–32
Monobactam	Aztreonam[c]	X		30	21–24	
Co-trimoxazole	Sulfamide[d]		X	300	13–17	
	Trimethoprim		X	5	15–18	
	Sulfamethoxazole/trimethoprim	X	X	23.75/1.25	13–16	
Tetracycline	Tetracycline[d]	X	X	30	12–15	
	Tigecycline		X	15	15–18	

Note: IPC = Institut Pasteur du Cambodge. IP-Paris = Institut Pasteur in Paris. MIC = MIC. All 103 human ESBL-*Ec* (88 colonization and 15 infection) were tested at IP-Paris by Kirby Bauer disk diffusion. All 93 fish and meat ESBL-*Ec* were tested at IPC by Kirby Bauer disk diffusion. Of these, 12/32 fish, 29/45 pork, and 8/16 chicken ESBL-*Ec* were additionally tested at IP-Paris by Kirby Bauer disk diffusion. MIC testing was only conducted at IP-Paris. Diameter and MIC interpretations were based on 2016 European Committee on Antimicrobial Susceptibility Testing recommendations unless otherwise noted.

[a]Diameters less than the lower bound were considered resistant. Diameters greater than or equal to the upper bound were considered susceptible. All other diamaters were considered intermediate.

[b]Growth at concentrations less than or equal to the lower bound were considered susceptible. Growth at concentrations greater than or equal to the upper bound were considered resistant.

[c]Only used at IPC to determine ESBL expression using the double-disk synergy test.

[d]Diameter and MIC interpretations based on 2016 Clinical and Laboratory Standards Institute recommendations.

Appendix Table 2. Characteristics of 150 fish, pork, and chicken samples purchased from two markets in Phnom Penh, Cambodia, 2016.

Source information	Fish N = 60 n(%)	Pork N = 60 n(%)	Chicken N = 30 n(%)
Market source			
Deum Kor	36(60)	36(60)	20(67)
Steung Meanchey	24(40)	24(40)	10(33)
Setting where animal raised[a,b]			
Large scale farm	29(48)	57(95)	23(77)
Backyard or village	6(10)	3(5)	7(23)
Wild	24(40)	0	0
Other types of meat/seafood sold at same stall where sample purchased[b,c]			
Yes	14(23)	2(3)	1(3)
No	45(75)	58(97)	27(90)

[a]Reported by meat vendors to the questionnaire administrator at time of purchase, but not independently verified.

[b]Totals may not sum to 100% due to missing information.

[c]Fish vendors sold other types of seafood or amphibians (e.g., crabs, shrimp, frogs), but never pork or chicken. Two pork vendors also sold chicken and one chicken vendor also sold pork.

Appendix Table 3. Characteristics and exposures among 88 healthy women colonized with ESBL-producing *Escherichia coli* in Phnom Penh, Cambodia, 2015–2016.

Characteristic	N = 88<br>n(%)
Age in years(mean, SD)	28(5)
Number of household members(mean, SD)	6(3)
Number of young children <5 y old(mean, SD)	2(1)
Hospitalized during pregnancy	
Yes	2 (2)
No	86 (98)
Antibiotics during pregnancy	
Yes	1 (1)
No	87 (99)
Location of recent childbirth	
Health center	35(40)
Hospital	31(35)
Private clinic	22(25)
Given antibiotics at birth[a]	
Yes	13(15)
No	71(81)
Unknown	4(5)
Birth by Cesarian section	
Yes	16(18)
No	72(82)
Drinking water treatment method[a]	
No treatment	12(14)
Boiling	41(47)
Disinfectant	14(16)
Filtration	8(9)
Other or unknown	13(15)
Toilet shared with neighboring households	
Yes	27(31)
No	61(69)
Pour flush toilet	
Yes	73(83)
No	15(17)
Contact with pets	
Yes	19(22)
No	69(78)
Contact with live poultry animals[a]	
Yes	11(13)
No	77(88)
Source of household meat and produce	
Steung Meanchey market	29(33)
Deum Kor market	3(3)
Neighborhood or street vendors[b]	56(64)
Pork consumption	
≥3/week	75(85)
<3/week	13(15)
Fish consumption[a]	
≥3/week	55(63)
<3/week	33(38)
Poultry consumption	
≥1/week	49(55)
<1/week	40(45)
Beef consumption	
≥1/week	18(20)
<1/week	70(80)
Dried pork consumption	
≥1/week	47(53)
<1/week	41(47)
Dried fish consumption	
≥1/week	25(28)
<1/week	63(72)
Dried poultry consumption	
Ever	66(75)
Never	22(25)
Dried beef consumption[a]	
Ever	55(63)
Never	33(38)

Characteristic	N = 88<br>n(%)
Raw vegetable consumption	
≥1/week	13(15)
<1/week	77(85)

[a]Totals may exceed 100% due to rounding.
[b]Neighborhood and street vendors purchased their produce each day from Deum Kor Market (Phnom Penh Hygiene, personal communication).

Appendix Table 4. Characteristics of 15 patients with ESBL-producing *Escherichia coli* infections presenting at the Sihanouk Center Hospital Center for Hope in Phnom Penh, Cambodia, between November 2015 and December 2016.

Characteristic	N = 15<br>n(%)
Age in years(median, SD)	64(13)
Female	11(73)
Infection type	
Blood	12(80)
Urine	2(13)
Peritoneal fluid	1(7)
Previous hospitalization <2 mo prior	2(13)
Infection detected ≥48 h after intake	2(13)

Appendix Table 5. Accession numbers for sequences of 196 ESBL-producing *Escherichia coli*, deposited in the European Nucleotide Archive under project number PRJEB25898.

Sample	Accession	Experiment	Source	Type	ST	ESBL Gene Type(s)
ERR2538560	ERS2367559	ERX2557097	Human	Fecal Swab	410	CTX-M-55
ERR2538133	ERS2367354	ERX2556670	Human	Fecal Swab	7584	CTX-M-27
ERR2538134	ERS2367355	ERX2556671	Human	Fecal Swab	3075	CTX-M-14
ERR2538135	ERS2367357	ERX2556672	Human	Fecal Swab	405	CTX-M-15
ERR2538136	ERS2367358	ERX2556673	Human	Fecal Swab	1722	CTX-M-15,CTX-M-27
ERR2538137	ERS2367359	ERX2556674	Human	Fecal Swab	38	CTX-M-15,CTX-M-27
ERR2538138	ERS2367360	ERX2556675	Human	Fecal Swab	871	CTX-M-14
ERR2538139	ERS2367361	ERX2556676	Human	Fecal Swab	3052	CTX-M-27,SHV-12
ERR2538140	ERS2367362	ERX2556677	Human	Fecal Swab	6438	CTX-M-14
ERR2538141	ERS2367363	ERX2556678	Human	Fecal Swab	155	CTX-M-15
ERR2538142	ERS2367364	ERX2556679	Human	Fecal Swab	5147	CTX-M-15
ERR2538143	ERS2367365	ERX2556680	Human	Fecal Swab	410	CTX-M-55
ERR2538144	ERS2367366	ERX2556681	Human	Fecal Swab	10	CTX-M-55
ERR2538145	ERS2367367	ERX2556682	Human	Fecal Swab	394	CTX-M-3
ERR2538146	ERS2367368	ERX2556683	Human	Fecal Swab	1722	CTX-M-14,CTX-M-55
ERR2538147	ERS2367369	ERX2556684	Human	Fecal Swab	156	CTX-M-15
ERR2538148	ERS2367370	ERX2556685	Human	Fecal Swab	10	CTX-M-27
ERR2538149	ERS2367371	ERX2556686	Human	Fecal Swab	394	CTX-M-15
ERR2538150	ERS2367373	ERX2556687	Human	Fecal Swab	410	CTX-M-15
ERR2538151	ERS2367374	ERX2556688	Human	Fecal Swab	10	CTX-M-55
ERR2538152	ERS2367375	ERX2556689	Human	Fecal Swab	156	CTX-M-15
ERR2538153	ERS2367376	ERX2556690	Human	Fecal Swab	156	CTX-M-55
ERR2538154	ERS2367377	ERX2556691	Human	Fecal Swab	4456	CTX-M-55
ERR2538155	ERS2367378	ERX2556692	Human	Fecal Swab	1081	CTX-M-14
ERR2538156	ERS2367379	ERX2556693	Human	Fecal Swab	131	CTX-M-15
ERR2538157	ERS2367380	ERX2556694	Human	Fecal Swab	6390	CTX-M-55
ERR2538158	ERS2367381	ERX2556695	Human	Fecal Swab	48	CTX-M-55
ERR2538159	ERS2367382	ERX2556696	Human	Fecal Swab	345	CTX-M-55
ERR2538160	ERS2367383	ERX2556697	Human	Fecal Swab	421	CTX-M-27
ERR2538161	ERS2367384	ERX2556698	Human	Fecal Swab	1588	CTX-M-15
ERR2538162	ERS2367385	ERX2556699	Human	Fecal Swab	1163	CTX-M-27
ERR2538163	ERS2367386	ERX2556700	Human	Fecal Swab	38	CTX-M-15
ERR2538164	ERS2367387	ERX2556701	Human	Fecal Swab	38	CTX-M-14
ERR2538165	ERS2367389	ERX2556702	Human	Fecal Swab	636	SHV-12
ERR2538166	ERS2367390	ERX2556703	Human	Fecal Swab	4040	CTX-M-15
ERR2538167	ERS2367391	ERX2556704	Human	Fecal Swab	10	CTX-M-55
ERR2538168	ERS2367392	ERX2556705	Human	Fecal Swab	345	CTX-M-55
ERR2538169	ERS2367393	ERX2556706	Human	Fecal Swab	1196	CTX-M-55
ERR2538170	ERS2367394	ERX2556707	Human	Fecal Swab	695	CTX-M-55
ERR2538171	ERS2367395	ERX2556708	Human	Fecal Swab	3052	CTX-M-15
ERR2538172	ERS2367396	ERX2556709	Human	Fecal Swab	155	CTX-M-15
ERR2538173	ERS2367397	ERX2556710	Human	Fecal Swab	405	CTX-M-15
ERR2538174	ERS2367398	ERX2556711	Human	Fecal Swab	405	CTX-M-15

Sample	Accession	Experiment	Source	Type	ST	ESBL Gene Type(s)
ERR2538175	ERS2367399	ERX2556712	Human	Fecal Swab	3052	CTX-M-15
ERR2538176	ERS2367400	ERX2556713	Human	Fecal Swab	10	CTX-M-15
ERR2538177	ERS2367401	ERX2556714	Human	Fecal Swab	3580	CTX-M-15
ERR2538178	ERS2367402	ERX2556715	Human	Fecal Swab	1656	CTX-M-55
ERR2538179	ERS2367403	ERX2556716	Human	Fecal Swab	162	CTX-M-15
ERR2538180	ERS2367405	ERX2556717	Human	Fecal Swab	131	CTX-M-27
ERR2538181	ERS2367406	ERX2556718	Human	Fecal Swab	10	CTX-M-55
ERR2538182	ERS2367407	ERX2556719	Human	Fecal Swab	421	CTX-M-27
ERR2538183	ERS2367408	ERX2556720	Human	Fecal Swab	7590	CTX-M-55
ERR2538184	ERS2367409	ERX2556721	Human	Fecal Swab	13	CTX-M-15
ERR2538185	ERS2367410	ERX2556722	Human	Fecal Swab	48	CTX-M-55
ERR2538186	ERS2367411	ERX2556723	Human	Fecal Swab	7590	CTX-M-55
ERR2538187	ERS2367412	ERX2556724	Human	Fecal Swab	8375	CTX-M-15
ERR2538188	ERS2367413	ERX2556725	Human	Fecal Swab	10	CTX-M-55
ERR2538189	ERS2367414	ERX2556726	Human	Fecal Swab	1244	CTX-M-14
ERR2538190	ERS2367415	ERX2556727	Human	Fecal Swab	38	CTX-M-15
ERR2538191	ERS2367416	ERX2556728	Human	Fecal Swab	131	CTX-M-15
ERR2538192	ERS2367417	ERX2556729	Human	Fecal Swab	410	CTX-M-15
ERR2538193	ERS2367418	ERX2556730	Human	Fecal Swab	617	CTX-M-15
ERR2538194	ERS2367419	ERX2556731	Human	Fecal Swab	405	CTX-M-15
ERR2538195	ERS2367420	ERX2556732	Human	Fecal Swab	5044	CTX-M-15
ERR2538196	ERS2367421	ERX2556733	Human	Fecal Swab	1722	CTX-M-55
ERR2538197	ERS2367423	ERX2556734	Human	Fecal Swab	131	CTX-M-27
ERR2538198	ERS2367424	ERX2556735	Human	Fecal Swab	969	CTX-M-27
ERR2538199	ERS2367425	ERX2556736	Human	Fecal Swab	10	CTX-M-15
ERR2538200	ERS2367426	ERX2556737	Human	Fecal Swab	215	CTX-M-15
ERR2538201	ERS2367427	ERX2556738	Human	Fecal Swab	421	CTX-M-27
ERR2538202	ERS2367428	ERX2556739	Human	Fecal Swab	43	CTX-M-15
ERR2539424	ERS2439626	ERX2557842	Human	Fecal Swab	6303	CTX-M-15
ERR2538203	ERS2367429	ERX2556740	Human	Fecal Swab	361	CTX-M-55
ERR2538204	ERS2367430	ERX2556741	Human	Fecal Swab	442	CTX-M-14
ERR2538205	ERS2367431	ERX2556742	Human	Fecal Swab	2083	CTX-M-55
ERR2538206	ERS2367432	ERX2556743	Human	Fecal Swab	226	CTX-M-15
ERR2538207	ERS2367433	ERX2556744	Human	Fecal Swab	2040	CTX-M-15
ERR2538208	ERS2367434	ERX2556745	Human	Fecal Swab	38	CTX-M-14
ERR2539425	ERS2439625	ERX2557843	Human	Fecal Swab	7160	CTX-M-55
ERR2538209	ERS2367435	ERX2556746	Human	Fecal Swab	2003	CTX-M-55
ERR2538210	ERS2367436	ERX2556747	Human	Fecal Swab	155	CTX-M-55
ERR2538211	ERS2367437	ERX2556748	Human	Fecal Swab	38	CTX-M-15
ERR2538212	ERS2367438	ERX2556749	Human	Fecal Swab	13	CTX-M-15
ERR2538213	ERS2367439	ERX2556750	Food	Poultry	10	CTX-M-55
ERR2538214	ERS2367440	ERX2556751	Food	Poultry	457	CTX-M-27
ERR2538215	ERS2367442	ERX2556752	Food	Poultry	48	CTX-M-55
ERR2538216	ERS2367443	ERX2556753	Food	Poultry	5713	CTX-M-14
ERR2538217	ERS2367444	ERX2556754	Food	Poultry	1844	CTX-M-55
ERR2538218	ERS2367445	ERX2556755	Food	Poultry	602	CTX-M-55
ERR2538219	ERS2367446	ERX2556756	Food	Poultry	10	CTX-M-55
ERR2538220	ERS2367447	ERX2556757	Food	Poultry	2705	CTX-M-55
ERR2538221	ERS2367448	ERX2556758	Food	Poultry	602	CTX-M-55
ERR2538222	ERS2367449	ERX2556759	Food	Poultry	3873	CTX-M-15
ERR2538223	ERS2367450	ERX2556760	Food	Poultry	3014	CTX-M-55
ERR2538224	ERS2367451	ERX2556761	Food	Poultry	7369	CTX-M-55
ERR2538225	ERS2367452	ERX2556762	Food	Poultry	2207	CTX-M-55
ERR2538226	ERS2367453	ERX2556763	Food	Poultry	155	CTX-M-55
ERR2538227	ERS2367454	ERX2556764	Food	Fish	156	CTX-M-15
ERR2538228	ERS2367455	ERX2556765	Food	Fish	1290	CTX-M-55
ERR2538229	ERS2367456	ERX2556766	Food	Fish	7585	CTX-M-55
ERR2538230	ERS2367457	ERX2556767	Food	Fish	424	CTX-M-55
ERR2538231	ERS2367459	ERX2556768	Food	Fish	6706	CTX-M-55
ERR2538232	ERS2367460	ERX2556769	Food	Fish	746	CTX-M-55
ERR2538233	ERS2367461	ERX2556770	Food	Fish	155	CTX-M-55
ERR2538234	ERS2367462	ERX2556771	Food	Fish	515	CTX-M-14
ERR2538235	ERS2367463	ERX2556772	Food	Fish	58	CTX-M-15
ERR2538236	ERS2367464	ERX2556773	Food	Fish	48	CTX-M-55
ERR2538237	ERS2367465	ERX2556774	Food	Fish	156	CTX-M-55
ERR2538238	ERS2367466	ERX2556775	Food	Fish	8399	CTX-M-55
ERR2538239	ERS2367467	ERX2556776	Food	Fish	7586	CTX-M-65
ERR2538240	ERS2367468	ERX2556777	Food	Fish	206	CTX-M-55
ERR2538241	ERS2367469	ERX2556778	Food	Fish	10	CTX-M-24

Sample	Accession	Experiment	Source	Type	ST	ESBL Gene Type(s)
ERR2538242	ERS2367470	ERX2556779	Food	Fish	7370	CTX-M-55
ERR2538243	ERS2367471	ERX2556780	Food	Fish	1485	CTX-M-55
ERR2538244	ERS2367472	ERX2556781	Food	Fish	1266	CTX-M-55
ERR2538245	ERS2367473	ERX2556782	Food	Fish	224	CTX-M-55
ERR2538246	ERS2367475	ERX2556783	Food	Fish	3873	CTX-M-15
ERR2538247	ERS2367476	ERX2556784	Food	Fish	195	CTX-M-14
ERR2538248	ERS2367477	ERX2556785	Food	Fish	2207	CTX-M-65
ERR2538249	ERS2367478	ERX2556786	Food	Fish	2690	CTX-M-55
ERR2538250	ERS2367479	ERX2556787	Food	Fish	1196	CTX-M-55
ERR2538251	ERS2367480	ERX2556788	Food	Fish	2690	CTX-M-55
ERR2538252	ERS2367481	ERX2556789	Food	Fish	5834	CTX-M-55
ERR2538253	ERS2367482	ERX2556790	Food	Fish	10	CTX-M-55
ERR2538254	ERS2367483	ERX2556791	Food	Fish	744	CTX-M-55
ERR2538255	ERS2367484	ERX2556792	Food	Pork	101	CTX-M-27
ERR2538256	ERS2367485	ERX2556793	Food	Pork	7589	CTX-M-55
ERR2538257	ERS2367486	ERX2556794	Food	Pork	641	CTX-M-55
ERR2538258	ERS2367487	ERX2556795	Food	Pork	617	CTX-M-55
ERR2538259	ERS2367488	ERX2556796	Food	Pork	6799	CTX-M-55
ERR2538260	ERS2367489	ERX2556797	Food	Pork	540	CTX-M-55
ERR2538261	ERS2367490	ERX2556798	Food	Pork	58	CTX-M-55
ERR2538262	ERS2367491	ERX2556799	Food	Pork	165	CTX-M-14
ERR2538263	ERS2367492	ERX2556800	Food	Pork	457	CTX-M-27
ERR2538264	ERS2367494	ERX2556801	Food	Pork	155	CTX-M-27
ERR2538265	ERS2367495	ERX2556802	Food	Pork	7588	CTX-M-14
ERR2538266	ERS2367496	ERX2556803	Food	Pork	746	CTX-M-55
ERR2538267	ERS2367497	ERX2556804	Food	Pork	1408	CTX-M-15
ERR2538268	ERS2367498	ERX2556805	Food	Pork	542	CTX-M-14
ERR2538269	ERS2367499	ERX2556806	Food	Pork	58	CTX-M-55
ERR2538270	ERS2367500	ERX2556807	Food	Pork	48	CTX-M-14
ERR2538271	ERS2367501	ERX2556808	Food	Pork	278	CTX-M-55
ERR2538327	ERS2367502	ERX2556864	Food	Pork	4956	CTX-M-55
ERR2538328	ERS2367503	ERX2556865	Food	Pork	515	CTX-M-14
ERR2538329	ERS2367504	ERX2556866	Food	Pork	8401	CTX-M-55
ERR2538330	ERS2367505	ERX2556867	Food	Pork	542	CTX-M-14
ERR2538331	ERS2367506	ERX2556868	Food	Pork	515	CTX-M-14
ERR2538332	ERS2367507	ERX2556869	Food	Pork	1081	CTX-M-14
ERR2538333	ERS2367508	ERX2556870	Food	Pork	540	CTX-M-55
ERR2538334	ERS2367509	ERX2556871	Food	Pork	101	CTX-M-14
ERR2538335	ERS2367510	ERX2556872	Food	Pork	58	CTX-M-55
ERR2538336	ERS2367512	ERX2556873	Food	Pork	1237	CTX-M-55
ERR2538337	ERS2367513	ERX2556874	Food	Pork	195	CTX-M-14
ERR2538338	ERS2367514	ERX2556875	Food	Pork	641	CTX-M-14
ERR2538339	ERS2367515	ERX2556876	Food	Pork	540	CTX-M-55
ERR2538340	ERS2367516	ERX2556877	Food	Pork	7589	CTX-M-55
ERR2538341	ERS2367517	ERX2556878	Food	Pork	1139	CTX-M-55
ERR2538342	ERS2367518	ERX2556879	Food	Pork	398	CTX-M-55
ERR2538343	ERS2367519	ERX2556880	Food	Pork	1258	CTX-M-15
ERR2538344	ERS2367520	ERX2556881	Food	Pork	354	CTX-M-55
ERR2538345	ERS2367521	ERX2556882	Food	Pork	398	CTX-M-14
ERR2538346	ERS2367522	ERX2556883	Food	Pork	744	CTX-M-55
ERR2538347	ERS2367523	ERX2556884	Food	Pork	4417	CTX-M-55
ERR2538348	ERS2367524	ERX2556885	Food	Pork	10	CTX-M-55
ERR2538545	ERS2367543	ERX2557082	Human	Urine	410	CTX-M-15
ERR2538546	ERS2367544	ERX2557083	Human	Urine	410	CTX-M-15
ERR2538547	ERS2367545	ERX2557084	Human	Blood	393	CTX-M-55
ERR2538548	ERS2367547	ERX2557085	Human	Blood	131	CTX-M-27
ERR2538549	ERS2367548	ERX2557086	Human	Blood	2011	CTX-M-15
ERR2538550	ERS2367549	ERX2557087	Human	Peritoneal	131	CTX-M-27
ERR2538551	ERS2367550	ERX2557088	Human	Blood	4456	CTX-M-55
ERR2538552	ERS2367551	ERX2557089	Human	Blood	131	CTX-M-27
ERR2538553	ERS2367552	ERX2557090	Human	Blood	131	CTX-M-14
ERR2538554	ERS2367553	ERX2557091	Human	Blood	12	CTX-M-14
ERR2538555	ERS2367554	ERX2557092	Human	Blood	1193	CTX-M-27
ERR2538556	ERS2367555	ERX2557093	Human	Blood	131	CTX-M-15
ERR2538557	ERS2367556	ERX2557094	Human	Blood	405	CTX-M-15
ERR2538558	ERS2367557	ERX2557095	Human	Blood	131	CTX-M-14
ERR2538559	ERS2367558	ERX2557096	Human	Blood	131	CTX-M-15
ERR2538528	ERS2367525	ERX2557065	Food	Poultry	6962	CTX-M-55
ERR2538529	ERS2367526	ERX2557066	Food	Poultry	5855	CTX-M-27

Sample	Accession	Experiment	Source	Type	ST	ESBL Gene Type(s)
ERR2538530	ERS2367527	ERX2557067	Food	Fish	189	CTX-M-55
ERR2538531	ERS2367529	ERX2557068	Food	Fish	189	CTX-M-55
ERR2538532	ERS2367530	ERX2557069	Food	Fish	3268	CTX-M-15
ERR2538533	ERS2367531	ERX2557070	Food	Fish	871	CTX-M-55
ERR2538534	ERS2367532	ERX2557071	Human	Fecal Swab	6361	CTX-M-15
ERR2538535	ERS2367533	ERX2557072	Human	Fecal Swab	6361	CTX-M-15
ERR2538536	ERS2367534	ERX2557073	Food	Pork	8377	CTX-M-55
ERR2538537	ERS2367535	ERX2557074	Human	Fecal Swab	189	CTX-M-55
ERR2538538	ERS2367536	ERX2557075	Human	Fecal Swab	6361	CTX-M-15
ERR2538539	ERS2367537	ERX2557076	Human	Fecal Swab	101	CTX-M-15
ERR2538540	ERS2367538	ERX2557077	Food	Pork	4956	CTX-M-55
ERR2538541	ERS2367539	ERX2557078	Food	Pork	195	CTX-M-14
ERR2538542	ERS2367540	ERX2557079	Food	Pork	2345	CTX-M-55
ERR2538543	ERS2367541	ERX2557080	Food	Pork	8400	CTX-M-55
ERR2538544	ERS2367542	ERX2557081	Food	Pork	8262	CTX-M-55

Note: ST = Multilocus sequence type. ESBL = Extended-spectrum β lactamase.

Appendix Table 6. Antibiotic resistance profiles of 93 ESBL-producing *Escherichia coli* from meat and fish purchased from markets and the distribution of acquired resistance genes encoding these phenotypes, Phnom Penh, Cambodia, 2015–2016.

Antibiotic Class	Resistant Phenotype and Detected Genes[a,b]	Fish N = 32 n(%)	Pork N = 45 n(%)	Chicken N = 16 n(%)	*p*-value[c]
**Third-generation cephalosporin**	**Resistant Phenotype**	32(100)	45(100)	16(100)	1.00
	**Detected Genes**				
	$bla_{CTX-M-55}$	23(72)	27(60)	12(75)	
	$bla_{CTX-M-14}$	2(6)	13(29)	1(6)	
	$bla_{CTX-M-15}$	4(13)	2(4)	1(6)	
	$bla_{CTX-M-24}$	1(3)	0	0	
	$bla_{CTX-M-27}$	0	3(7)	2(13)	
	$bla_{CTX-M-65}$	2(6)	0	0	
	bla_{CMY-2}	1(3)	1(2)	0	
**Aminoglycoside**	**Resistant Phenotype**	26(81)	42(93)	12(75)	0.12
	**Detected Genes**				
	aph(3′)-Ia	16(50)	11(24)	6(38)	
	strA	11(34)	14(31)	8(50)	
	strB	21(66)	16(36)	10(63)	
	aadA1	9(28)	12(27)	1(6)	
	aadA2	10(31)	27(60)	4(25)	
	aadA22	5(16)	0	2(13)	
	aac(3)-IId	18(54)	27(60)	6(38)	
	aac(6')Ib-cr	3(9)	0	1(6)	
**Amphenicol**	**Resistant Phenotype[f]**	26(81)	40(89)	11(69)	0.18
	**Detected Genes**				
	catA1	3(9)	0	1(6)	
	catA2	5(16)	6(13)	1(6)	
	floR	17(53)	27(60)	9(56)	
	cmlA	6(19)	25(56)	2(13)	
**Carbapenem**	**Resistant Phenotype[d]**	1(3)	0	0	
	**Detected Genes**				
	$bla_{OXA-181}$	1(3)	0	0	
**Colistin**	**Resistant Phenotype**	3(9)	8(18)	0	0.16
	**Detected Genes[e]**				
	mcr1	3(9)	6(13)	0	
	mcr3	0	3(7)	0	
**Fluoroquinolone**	**Resistant Phenotype**	28(88)	32(71)	14(88)	0.15
	**Detected Genes[e]**				

	qnrS1	26(81)	33(73)	12(75)	
	aac(6')Ib-cr	3(9)	0	1(6)	
	oqxA	0	0	1(6)	
**Macrolide**	**Resistant Phenotype[f]**	22(69)	26(58)	8(50)	0.42
	**Detected Genes**				
	erm(B)	3(9)	4(9)	2(13)	
	mph(A)	17(53)	10(22)	7(44)	
	mef(B)	4(13)	18(40)	0	
	lnu(F)	14(44)	5(11)	4(25)	
**Sulphamethoxazole/ Trimethoprim**	**Resistant Phenotype**	28(88)	39(87)	13(81)	0.83
	**Detected Genes[e]**				
	sul1	9(28)	0	4(25)	
	sul2	17(53)	23(51)	9(56)	
	sul3	17(53)	32(71)	4(25)	
	dfrA12	7(22)	30(67)	2(13)	
	dfrA14	20(63)	8(18)	8(50)	
	dfrA17	3(9)	1(2)	2(13)	
**Tetracycline**	**Resistant Phenotype**	28(88)	41(91)	14(88)	0.85
	**Detected Genes**				
	tet(A)	26(81)	39(87)	13(81)	
	tet(B)	3(9)	4(9)	1(6)	
	tet(M)	2(6)	23(51)	1(6)	

Note: ESBL = Extended-spectrum β lactamase. All 93 isolates produced ESBLs; 2/93 additionally produced Amp-C β lactamases (CMY-type).
[a]The frequency of resistance genes detected may exceed the total number of isolates exhibiting resistance to a given antibiotic class because many isolates carried multiple genes encoding resistance to the same antibiotic class.
[b]Isolates were categorized as "Resistant" if they demonstrated intermediate or complete phenotypic resistance to any antibiotic within the stated class.
[c]*p*-values were generated using Fisher exact tests comparing the distributions of phenotypic antibiotic resistance patterns between samples types.
[d]We recovered an additional carbapanemase-producing (OXA-48), non-ESBL producing *E. coli* from one pork sample (data not shown here).
[e]Frequency of detected resistance genes may not sum to total number of isolates exhibiting resistant phenotype. Some resistance phenotypes may be encoded by point mutations, but these were not investigated.
[f]Phenotypic resistance to this antibiotic class was assessed for 49/93 ESBL-*Ec* isolates. For 20/32 ESBL-*Ec* from fish, 16/45 from pork, and 8/16 from poultry, phenotypic resistance is reported based on the occurrence of one of more genes conferring resistance to this antibiotic class.

Appendix Table 7. Antibiotic resistance profiles of ESBL-producing *Escherichia coli* from 88 healthy, colonized humans and 15 infected patients, and the distribution of acquired resistance genes encoding these phenotypes, Phnom Penh, Cambodia, 2015–2016.

Antibiotic Class	Resistant Phenotype and Detected Genes[a,b]	Colonization N = 88 n(%)	Clinical N = 15 n(%)	*p*-value[c]
**Third-generation cephalosporin**	**Resistant Phenotype**	88(100)	15(100)	1.00
	**Detected Genes**			
	$bla_{\text{CTX-M-3}}$	1(1)	0	
	$bla_{\text{CTX-M-55}}$	27(31)	2(13)	
	$bla_{\text{CTX-M-14}}$	9(10)	3(20)	
	$bla_{\text{CTX-M-15}}$	41(47)	6(40)	
	$bla_{\text{CTX-M-27}}$	13(15)	4(27)	
	$bla_{\text{CMY-2}}$	3(3)	3(20)	
	$bla_{\text{CMY-42}}$	3(3)	0	
	$bla_{\text{SHV-12}}$	2(2)	0	
**Aminoglycoside**	**Resistant Phenotype**	75(85)	14(93)	0.69
	**Detected Genes**			
	aph(3')-Ia	10(11)	1(7)	
	strA	43(49)	10(67)	
	strB	42(48)	10(67)	
	aadA1	7(8)	0	
	aadA2	20(23)	1(7)	
	aadA5	39(44)	12(80)	

Antibiotic Class	Resistant Phenotype and Detected Genes[a,b]	Colonization N = 88 n(%)	Clinical N = 15 n(%)	*p*-value[c]
	aadA22	3(3)	0	
	aac(3)-IId	32(36)	4(27)	
	aac(3)-IIa	15(17)	5(33)	
	aac(6')Ib-cr	12(14)	6(40)	
**Amphenicol**	**Resistant Phenotype[d]**	29(33)	1(7)	0.06
	**Detected Genes**			
	catA1	7(8)	0	
	catA2	8(9)	1(7)	
	floR	15(17)	0	
	cmlA	6(7)	0	
**Carbapenem**	**Resistant Phenotype**	3(3)	3(20)	0.04
	**Detected Genes**			
	bla_{NDM-1}	1(1)	0	
	bla_{NDM-5}	2(2)	1(7)	
	$bla_{OXA-181}$	0	1(7)	
**Colistin**	**Resistant Phenotype**	2(2)	0	
	**Detected Genes**			
	mcr1	1(1)	0	
	mcr3	1(1)	0	
**Fluoroquinolone**	**Resistant Phenotype[d]**	86(98)	15(100)	1.00
	**Detected Genes**			
	qnrS1	46(52)	1(7)	
	aac(6')Ib-cr	12(14)	6(40)	
**Macrolide**	**Resistant Phenotype[d]**	40(45)	12(80)	0.02
	**Detected Genes**			
	erm(B)	6(7)	2(13)	
	mph(A)	42(49)	12(80)	
	mef(B)	5(6)	0	
	lnu(F)	9(10)	1(7)	
**Sulfonamide/ Trimethoprim**	**Resistant Phenotype**	75(85)	13(87)	1.00
	**Detected Genes**			
	sul1	45(51)	12(80)	
	sul2	47(53)	10(67)	
	sul3	15(17)	1(7)	
	dfrA1	4(5)	0	
	dfrA12	18(20)	1(7)	
	dfrA14	22(25)	1(7)	
	dfrA17	38(43)	12(80)	
**Tetracycline**	**Resistant Phenotype[d]**	71(81)	14(93)	0.46
	**Detected Genes**			
	tet(A)	49(56)	10(67)	
	tet(B)	27(31)	4(27)	
	tet(D)	2(2)	0	
	tet(M)	5(6)	0	

Note: ESBL = Extended-spectrum β lactamase. All 103 isolates produced ESBLs; 9/103 additionally produced Amp-C β lactamases (CMY-type).
[a]The frequency of resistance genes detected may exceed the total number of isolates exhibiting resistance to a given antibiotic class because some isolates carried multiple genes encoding resistance to the same antibiotic class.
[b]Isolates were categorized as “Resistant” if they demonstrated intermediate or complete phenotypic resistance to any antibiotic within the stated class.
[c]*p*-values were generated using Fisher exact tests comparing the distributions of phenotypic antibiotic resistance patterns between samples types.
[d]Frequency of detected resistance genes may not sum to total number of isolates exhibiting resistant phenotype. Some resistance phenotypes may be encoded by point mutations, but these were not investigated.

Appendix Table 8. Multilocus sequence types of ESBL-producing *Escherichia coli* detected among humans and food in Phnom Penh, Cambodia, by phylogenetic clan.

Clan	MLST CC[a,b]	ST	Human colonization n = 35 (%)	Human infection n = 13 (%)	Meat n = 5 (%)	β-lactamase gene type(s) detected	Phylo-type[c]
Clan I/B2&D	38	–	10 (29)	0	1 (20)		–
n = 53		38	6 (17)	0	0	CTX-M-14 (2), CTX-M-15 (4), CTX-M-27 (1)	D
		2003	1 (3)	0	0	CTX-M-55 (1)	D
		3052	3 (9)	0	0	CTX-M-15 (2), CTX-M-27 (1), SHV-12 (1)	D
		3268	0	0	1 (20)	CTX-M-15 (1)	D
	Singletons	12	0	1 (8)	0	CTX-M-14 (1)	B2
		131	4 (11)	7 (54)	0	CTX-M-14 (2), CTX-M-15 (4), CTX-M-27 (5), CMY-2 (1)	B2/D
		354	0	0	1 (20)	CTX-M-55 (1)	D
		393	0	1 (8)	0	CTX-M-55 (1)	D
		394	2 (6)	0	0	CTX-M-3 (1), CTX-M-15 (1), CMY-2 (1)	D
		405	4 (11)	1 (8)	0	CTX-M-15 (5), NDM-5 (1)	D
		421	3 (9)	0	0	CTX-M-27 (3)	B2
		457	0	0	2 (40)	CTX-M-27 (2)	D
		636	1 (3)	0	0	SHV-12 (1)	D
		969	1 (3)	0	0	CTX-M-27 (1)	B2
		1163	1 (3)	0	0	CTX-M-27 (1), CMY-2 (1)	D
		1193	0	1 (8)	0	CTX-M-27 (1), CTX-M-55 (1)	B2
		1485	0	0	1 (20)	CTX-M-55 (1)	D
		1588	1 (3)	0	0	CTX-M-15 (1)	D
		1722	3 (9)	0	0	CTX-M-14 (1), CTX-M-15 (1), CTX-M-27 (1), CTX-M-55 (2)	D
		2011	0	1 (8)	0	CTX-M-15 (1)	D
		4040	1 (3)	0	0	CTX-M-15 (1)	D
		4456	1 (3)	1 (8)	0	CTX-M-55 (2)	B2
		5147	1 (3)	0	0	CTX-M-15 (1)	D
		6303	1 (3)	0	0	CTX-M-15 (1)	D
		8375	1 (3)	0	0	CTX-M-15 (1)	D
	MLST CC[a,b]	ST	Human colonization n = 20 (%)	Human infection n = 0 (%)	Meat n = 49 (%)		Phylo-type[c]
Clan II/A	10		12 (60)	0	11 (22)		–
n = 69		10	8 (40)	0	5 (10)	CTX-M-15 (2), CTX-M-55(9), CTX-M-27 (1), OXA-181 (1), CTX-M-24 (1)	A
		43	1 (5)	0	0	CTX-M-15 (1)	A
		48	2 (10)	0	3 (6)	CTX-M-14 (1), CTX-M-55 (4)	A
		215	1 (5)	0	0	CTX-M-15 (1)	A
		744	0	0	2 (4)	CTX-M-55 (2)	A
		5713	0	0	1 (2)	CTX-M-14 (1)	A
	Singletons	189	0	0	2 (4)	CTX-M-55 (2)	A
		195	0	0	3 (6)	CTX-M-14 (3)	A
		206	0	0	1 (2)	CTX-M-55 (1)	A
		361	1 (5)	0	0	CTX-M-55 (1)	A
		398	0	0	2 (4)	CTX-M-14 (1), CTX-M-55 (1)	A
		540	0	0	3 (6)	CTX-M-55 (3)	A
		542	0	0	2 (4)	CTX-M-14 (2)	A
		617	1 (5)	0	1 (2)	CTX-M-15 (1), CTX-M-55 (1), CMY-42 (1)	A
		695	1 (5)	0	0	CTX-M-55 (1)	A
		746	0	0	2 (4)	CTX-M-55 (2), CMY-2 (2)	A
		871	1 (5)	0	1 (2)	CTX-M-14 (1)	A
		1139	0	0	1 (2)	CTX-M-55 (1)	A

Clan	MLST CC[a,b]	ST	Human colonization n = 35 (%)	Human infection n = 13 (%)	Meat n = 5 (%)	β-lactamase gene type(s) detected	Phylo-type[c]
		1244	1 (5)	0	0	CTX-M-14 (1)	A
		1266	0	0	1 (2)	CTX-M-55 (1)	B2
		1290	0	0	1 (2)	CTX-M-55 (1)	A
		1408	0	0	1 (2)	CTX-M-15 (1)	A
		2207	0	0	2 (4)	CTX-M-55 (1), CTX-M-65 (1)	A
		2345	0	0	1 (2)	CTX-M-55 (1)	A
		2690	0	0	2 (4)	CTX-M-55 (2)	A
		2705	0	0	1 (2)	CTX-M-55 (1)	A
		3014	0	0	1 (2)	CTX-M-55 (1)	A
		3075	1 (5)	0	0	CTX-M-14 (1)	A
		5834	0	0	1 (2)	CTX-M-55 (1)	A
		5855	0	0	1 (2)	CTX-M-27 (1)	A
		6390	1 (5)	0	0	CTX-M-55 (1)	A
		6438	1 (5)	0	0	CTX-M-14 (1)	A
		6706	0	0	1 (2)	CTX-M-55 (1)	A
		7369	0	0	1 (2)	CTX-M-55 (1)	A
		7370	0	0	1 (2)	CTX-M-55 (1)	A
		7585	0	0	1 (2)	CTX-M-55 (1)	A
		7588	0	0	1 (2)	CTX-M-14 (1)	A
		7589	0	0	2 (4)	CTX-M-55 (2)	A
		8377	0	0	1 (2)	CTX-M-55 (1)	A
	MLST CC[a,b]	ST	Human colonization n = 19 (%)	Human infection n = 0 (%)	Meat n = 28 (%)		Phylo-type[c]
Clan III/B1 n = 47	156		3 (16)	0	4 (14)		–
		156	3 (16)	0	2 (7)	CTX-M-15 (3), CTX-M-55 (2)	B1
		3873	0	0	2 (7)	CTX-M-15 (2)	B1
	58		3 (16)	0	7 (25)		–
		58	0	0	4 (14)	CTX-M-15 (1), CTX-M-55 (3)	B1
		155	3 (16)	0	3 (11)	CTX-M-15 (2), CTX-M-27(1), CTX-M-55 (3)	B1
	Singletons	13	2 (11)	0	0	CTX-M-15 (2)	B1
		101	1 (5)	0	2 (7)	CTX-M-14 (1), CTX-M-15(1), CTX-M-27 (1), NDM-1 (1)	B1/Unknown
		162	1 (5)	0	0	CTX-M-15 (1)	B1
		224	0	0	1 (4)	CTX-M-55 (1)	B1
		278	0	0	1 (4)	CTX-M-55 (1)	B1
		345	2 (11)	0	0	CTX-M-55 (2)	B1
		424	0	0	1 (4)	CTX-M-55 (1)	B1
		442	1 (5)	0	0	CTX-M-14 (1)	B1
		602	0	0	2 (7)	CTX-M-55 (2)	B1
		641	0	0	1 (4)	CTX-M-14 (1), CTX-M-55 (1)	B1
		1081	1 (5)	0	1 (4)	CTX-M-14 (2)	B1
		1196	1 (5)	0	1 (4)	CTX-M-55 (2)	B1
		1258	0	0	1 (4)	CTX-M-15 (1)	B1
		2040	1 (5)	0	0	CTX-M-15 (1)	A
		3580	1 (5)	0	0	CTX-M-15 (1)	B1
		4956	0	0	2 (7)	CTX-M-55 (2)	B1
		6799	0	0	1 (4)	CTX-M-55 (1)	B1
		7586	0	0	1 (4)	CTX-M-65 (1)	B1
		7590	2 (11)	0	0	CTX-M-55 (2)	B1
		8399	0	0	1 (4)	CTX-M-55 (1)	B1
		8401	0	0	1 (4)	CTX-M-55 (1)	B1
	MLST CC[a,b]	ST	Human colonization n = 14 (%)	Human infection n = 2 (%)	Meat n = 11 (%)		Phylo-type[c]
Did not group in a clan[d] n = 27	Singletons	165	0	0	1 (9)	CTX-M-14 (1)	A
		189	1 (7)	0	0	CTX-M-55 (1)	A
		226	1 (7)	0	0	CTX-M-15 (1)	A

Clan	MLST CC[a,b]	ST	Human colonization n = 35 (%)	Human infection n = 13 (%)	Meat n = 5 (%)	β-lactamase gene type(s) detected	Phylo-type[c]
		410	4 (29)	2 (100)	0	CTX-M-15 (4), CTX-M-27(1) CTX-M-55 (2), NDM-5 (2), OXA-181 (1), CMY-42 (1)	A
		515	0	0	3 (27)	CTX-M-14 (3)	B1
		641	0	0	1 (9)	CTX-M-14 (1)	B1
		1237	0	0	1 (9)	CTX-M-55 (1)	A
		1656	1 (7)	0	0	CTX-M-55 (1)	B1
		1844	0	0	1 (9)	CTX-M-55 (1)	B1
		2083	1 (7)	0	0	CTX-M-55 (1)	B1
		4417	0	0	1 (9)	CTX-M-55 (1)	B1
		5044	1 (7)	0	0	CTX-M-15 (1)	A
		6361	3 (21)	0	0	CTX-M-15 (3)	A
		6962	0	0	1 (9)	CTX-M-55 (1)	A
		7160	1 (7)	0	0	CTX-M-55 (1)	Unknown
		7584	1 (7)	0	0	CTX-M-27 (1)	A
		8262	0	0	1 (9)	CTX-M-55 (1)	D
		8400	0	0	1 (9)	CTX-M-55 (1)	D

Note: ESBL = Extended-spectrum β lactamase. MLST CC = Multilocus sequence type clonal complex. ST = Sequence type. Clans were based on a phylogenetic tree inferred from the pairwise evolutionary distances between assembled whole genome sequences (Figure 2 in the main text). Each clan comprised an exclusive subset of STs.

[a]For each MLST CC, the cumulative frequency (and percentage) of all sequence types belonging to that CC are presented in the first row, in which ST is described as "–."

[b]"Singletons" refers to STs that did not share ≥6/7 alleles with any other ST in this dataset.

[c]Phylo-types assigned using the Clermont scheme.

[d]Includes one colonization ESBL-*Ec* that was excluded from phylogenetic analysis due to insufficient quality.

Appendix Table 9. Environmental exposures and healthy women's colonization with ESBL-producing *Escherichia coli* belonging to Clan II/A or Clan III/B1 (versus Clan I/B2&D), Phnom Penh, Cambodia, 2015–2016.

	Clan I/B2&D(reference)[a] N = 35	Clan II/A[a] N = 20		Clan III/B1[a] N = 19	
Exposure	n(%)	n(%)	aOR$_1$[b] (95% CI)	n(%)	aOR$_2$[b] (95% CI)
People living in home					
>8	4(11)	5(25)	1.8(0.4–8.4)	2(11)	1.1(0.2–7.6)
6–8	12(34)	3(15)	0.4(0.1–1.6)	9(47)	1.7(0.5–5.7)
≤5	19(54)	12(60)	ref	8(42)	ref
Place of delivery					
Private clinic	9(26)	5(25)	0.9(0.2–3.4)	3(16)	0.9(0.2–4.6)
Hospital	12(34)	5(25)	0.6(0.2–2.4)	10(53)	2.2(0.6–8.0)
Health center	14(40)	10(50)	ref	6(32)	ref
Antibiotics at birth[c]	6(17)	2(10)	0.6(0.1–3.2)	2(11)	0.6(0.1–3.3)
Untreated drinking water	3(9)	5(25)	3.4(0.7–16.3)	3(16)	1.9(0.3–10.6)
Toilet shared[d]	10(29)	6(30)	1.1(0.3–3.6)	6(32)	1.1(0.3–3.9)
Non-flush toilet	26(74)	18(90)	3.2(0.6–16.5)	15(79)	1.3(0.3–5.0)
Pet contact	7(20)	5(25)	1.5(0.4–5.7)	6(32)	2.1(0.6–7.8)
Live poultry contact	2(6)	4(20)	4.8(0.8–30.2)	4(21)	5.1(0.8–32.2)
Consumption habits					
Dry fish ≥1/week	6(17)	6(30)	2.4(0.6–9.3)	8(42)	4.2(1.1–16)
Dry pork ≥1/week	18(51)	13(65)	1.8(0.6–5.5)	11(58)	1.3(0.4–4.0)
Dry beef	23(66)	9(45)	0.4(0.1–1.3)	13(68)	1.1(0.3–3.8)
Dry poultry	27(77)	15(75)	0.9(0.2–3.1)	15(79)	1.1(0.3–4.2)
Shellfish	23(66)	11(55)	0.6(0.2–1.9)	17(89)	4.3(0.8–21.9)
Fish ≥3/week	25(71)	11(55)	0.5(0.2–1.6)	10(53)	0.5(0.1–1.5)
Pork ≥3/week	30(86)	17(85)	1(0.2–4.5)	17(89)	1.4(0.2–8.2)
Beef ≥1/week	7(20)	3(15)	0.7(0.2–3.2)	6(32)	1.9(0.5–6.8)
Poultry ≥1/week	16(46)	9(45)	1(0.3–3.0)	11(58)	1.6(0.5–5.1)
Insects	20(57)	12(60)	1.3(0.4–4)	11(58)	1.1(0.4–3.6)
Seafood	28(80)	14(70)	0.6(0.2–2.2)	16(84)	1.4(0.3–6.3)
Raw beef	11(31)	6(30)	0.8(0.2–2.9)	2(11)	0.2(0–1.2)
Raw veg ≥1/week	4(11)	3(15)	1.3(0.3–6.5)	3(16)	1.3(0.3–6.5)

Note: ESBL = Extended-spectrum β lactamase. aOR = Adjusted odds ratio. CI = Confidence interval.

[a]Overall N = 74. 14/88 human colonization ESBL-*Ec* were excluded: 1/14 was excluded from the minimum evolution phylogenetic tree due to insufficient quality; 13/14 did not group into a phylogenetic clan.

[b]Adjusted for age.

[c]Not reported for two women (missing data). One woman's colonization ESBL-*Ec* grouped in Clan I/B2&D while the other woman's grouped in Clan II/A.

[d]With other households.

5. Supplementary Figure

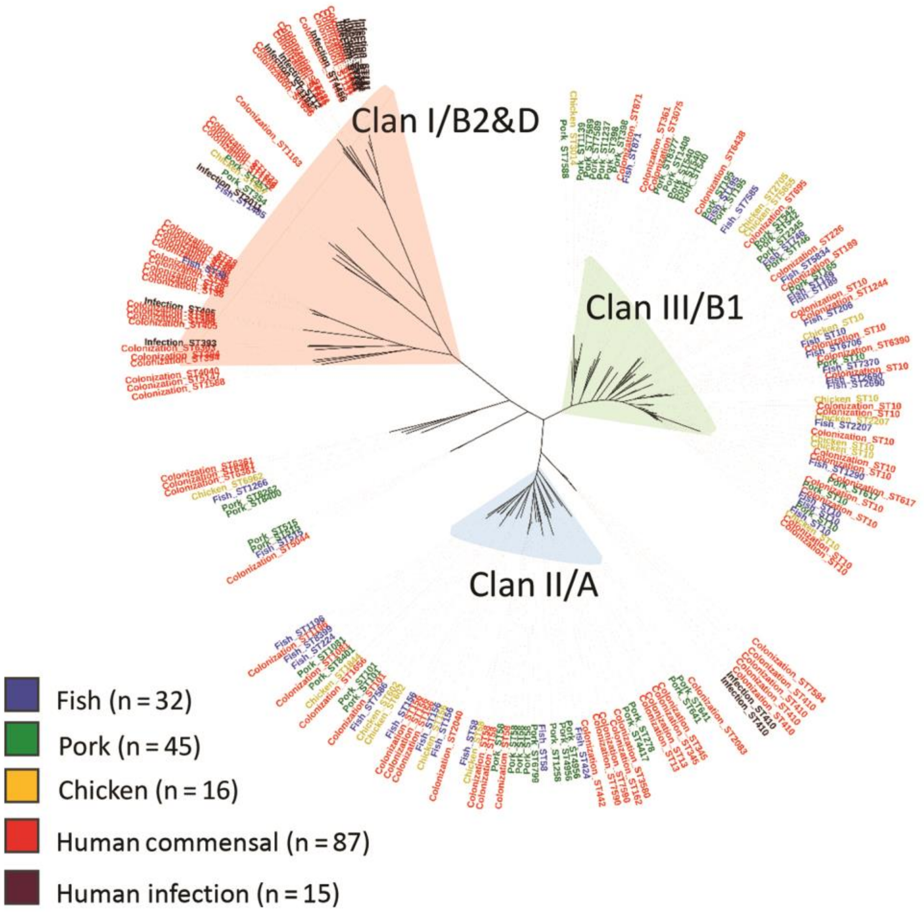

Appendix Figure. Core genome MLST-based phylogenetic tree of 195 ESBL-producing *E. coli* genomes comprising 87 human colonization isolates, 15 human clinical isolates and 93 isolates from fish, pork, and chicken meat, and resulting phylogenetic Clans I/B2&D (n = 53), II/A (n = 72), and III/B1 (n = 52).

Note: ESBL = Extended-spectrum β lactamase. ST = Sequence type.
